# Acute digital ischemia in a child following a suspected arthropod bite: A case report

**DOI:** 10.1097/MD.0000000000043286

**Published:** 2025-07-11

**Authors:** Abdallah A. Najjar, Mahmoud Abdelrazzaq Abu Mayaleh, Roba Alzuhoor, Mohamed Abdelrazzaq Abu Mayaleh, Fawzi Mazen Nejmah, Dalia Abu-Isneineh

**Affiliations:** a Faculty of Medicine, Hebron University, Hebron, Palestine; b Department of Pediatrics, Red Crescent Specialized Hospital, Hebron, Palestine; c Department of Pediatrics, Faculty of Medicine, Hebron University, Hebron, Palestine.

**Keywords:** acute limb ischemia, anticoagulation therapy, arthropod bite, conservative management, digital ischemia, localized vascular injury, multidisciplinary approach, pediatric ischemic events, pediatric vascular compromise, tissue perfusion

## Abstract

**Rationale:**

Acute limb ischemia is exceptionally rare in pediatric patients and poses a significant risk of permanent disability or limb loss. While arthropod bites are typically benign and self-limited, this case illustrates an unusual presentation of digital ischemia in a previously healthy toddler, suspected to be associated with an arthropod exposure. This report contributes to the limited literature highlighting vascular complications secondary to insect-related injuries in children.

**Patient concerns:**

A 2-year and 5-month-old male presented with a 3-day history of black discoloration in the second and third toes of the right foot, accompanied by erythema and localized tenderness.

**Diagnoses:**

There was no history of trauma, systemic illness, or recent vascular instrumentation. The child’s mother recalled observing an arthropod near the foot prior to symptom onset. Physical examination revealed ischemic changes localized to the affected toes without systemic involvement. Laboratory investigations revealed iron deficiency anemia with normal inflammatory and coagulation markers. Doppler ultrasound showed absent arterial flow in the plantar arch. A diagnosis of localized vascular compromise, potentially induced by an arthropod bite, was established based on clinical and imaging findings after exclusion of systemic causes.

**Intervention:**

The patient was treated conservatively with subcutaneous enoxaparin, low-dose aspirin, and prophylactic antibiotics. Surgical intervention was not required.

**Outcomes:**

Over the course of 30 days, the patient showed complete clinical recovery with full restoration of tissue perfusion.

**Lessons:**

This case emphasizes the importance of considering arthropod bite-induced vascular injury as a rare but significant differential diagnosis in pediatric digital ischemia. Early recognition and conservative management can lead to favorable outcomes and avoid invasive interventions.

## 1. Introduction

Acute limb ischemia (ALI) is defined as a sudden decrease in limb perfusion that threatens the viability of the affected extremity.^[1]^ While ALI is well-recognized in adults, primarily due to thromboembolic events in atherosclerotic arteries,^[2]^ its occurrence in pediatric populations is exceedingly rare, with an estimated incidence of 26 cases per 100,000 hospital admissions.^[3]^ The most commonly reported causes in children include iatrogenic injuries related to arterial cannulation or invasive procedures, followed by congenital anomalies and trauma.^[3–5]^

The clinical presentation of ALI in children differs significantly from adults and often necessitates a high index of suspicion. Among the less common but clinically significant causes is vascular injury secondary to arthropod exposure. Although most arthropod bites are benign and self-limiting, certain species can provoke exaggerated local reactions, vasospasm, or direct endothelial injury. Such responses may result in localized ischemia or necrosis.^[6,7]^ For instance, envenomation by the brown recluse spider *Loxosceles reclusa* has been shown to cause dermonecrotic lesions through neutrophil activation and vascular thrombosis, impairing endothelial function and inducing significant tissue injury.^[8]^

Recognition of these rare vascular complications is crucial, especially in the absence of trauma or systemic illness. This case report describes a toddler presenting with digital ischemia, suspected to be associated with arthropod exposure. It underscores the need for detailed clinical evaluation and timely multidisciplinary management. Moreover, the case demonstrates the potential for successful conservative therapy, aligning with existing evidence supporting nonoperative approaches in pediatric ALI.^[3,9]^

## 2. Case presentations

A 2-year and 5-month-old previously healthy male was referred to our tertiary care center with a 3-day history of black discoloration involving the second and third toes of the right foot, accompanied by erythema and localized tenderness. The child had no prior history of trauma, insect bites, vascular catheterization, or systemic illness. On further questioning, the mother reported observing an unidentified arthropod near the child’s foot shortly before symptom onset. No fever, rash, joint swelling, or systemic signs were noted.

The patient had an unremarkable past medical and surgical history. There were no known autoimmune or hematologic disorders, no prior hospitalizations, and no current medications. The family history was negative for thrombotic or vascular diseases.

**Figure 1.**
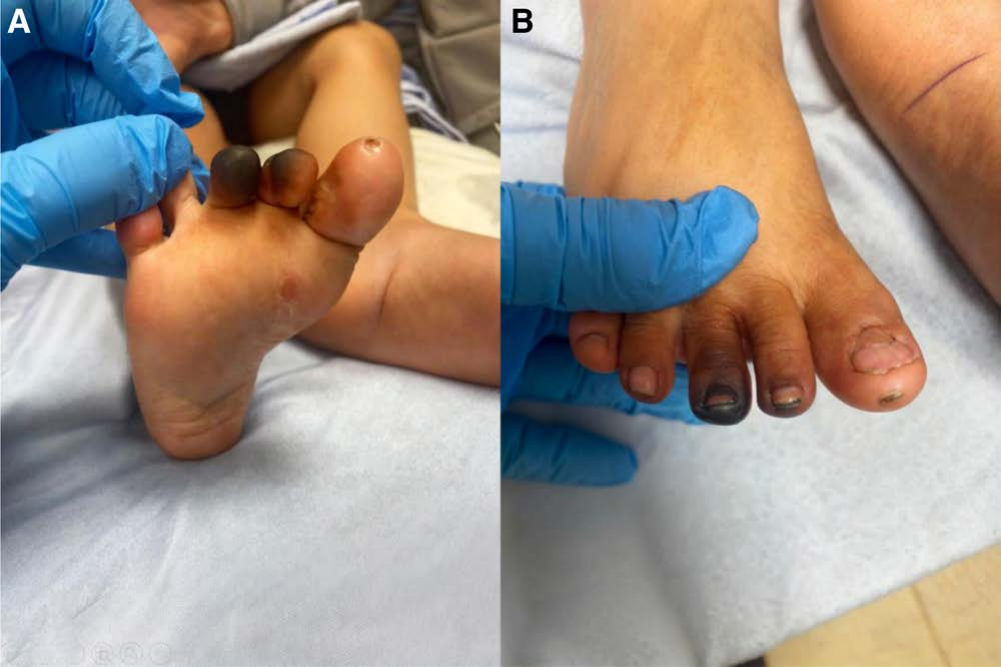
Clinical presentation of acute digital ischemia in the right foot. (A) Dorsal view of the right foot demonstrates blackish discoloration affecting the second and third toes, with intact surrounding skin and nails. The affected areas exhibit clear demarcation between viable and ischemic tissue, suggestive of vascular compromise. Mild erythema is noted at the base of the affected toes, consistent with inflammatory changes. (B) Plantar view of the right foot highlights the ischemic discoloration of the distal phalanges of the second and third toes. The toes exhibit darkened, necrotic-appearing tissue with no visible bullae, ulceration, or puncture marks. Adjacent skin appears minimally involved, with no overt signs of infection or secondary changes.

Upon presentation, the child was hemodynamically stable with the following vital signs: temperature 36.3°C, heart rate 107 bpm, respiratory rate 28 breaths/min, blood pressure 119/78 mm Hg, and oxygen saturation of 97% on room air. Physical examination revealed blackish discoloration of the second and third right toes with surrounding erythema, warmth, and tenderness (Fig. 1). Bilateral pedal pulses were palpable. Neurologic examination of the lower limbs was normal. Examination of the contralateral foot and other systemic findings were unremarkable.

Initial laboratory studies at the referring facility demonstrated mild microcytic anemia (hemoglobin 10.8 g/dL; MCV 65 fL) with low ferritin (7.3 ng/mL), suggestive of iron deficiency. Inflammatory markers, including CRP and ESR, were within normal limits. Coagulation profile, renal function tests, and infectious work-up were unremarkable (Tables 1–3).

**Table 1:** Summary of hematologic and inflammatory laboratory findings at presentation and during follow-up.

Parameter	Initial (day 1, local hospital)	Follow-up (day 4, our hospital)	Pediatric reference range	Comments
CBC				
Hemoglobin (g/dL)	10.8	11.06	11–13	Mild anemia with slight improvement
MCV (fL)	65	69.12	70–86	Persistent microcytosis
WBC (×10^9^/L)	7.3	10.81	6–17	Elevated in follow-up, possibly reactive
Neutrophils (%)	46	68.25	30–60	Neutrophilia in follow-up, consistent with inflammatory response
Lymphocytes (%)	41	22.78	40–70	Decreased in follow-up
Platelets (×10^9^/L)	376	345.2	150–450	Normal range, mild variation
Inflammatory markers				
CRP (mg/L)	N/A	6	<10	Normal in follow-up, no significant inflammation
ESR (mm/h)	20	10	0–10	Improved at follow-up, now normal
Coagulation studies				
PT (s)	12.8	13.7	11–14	Normal range
PTT (s)	40	24	25–35	Normalized
INR	0.9	1.05	0.8–1.2	Slightly elevated but clinically insignificant
D-dimer (ng/mL)	140	N/A	<500	Normal, no evidence of hypercoagulability
Blood film analysis				
Morphology	N/A	Normal morphology		Mild hypochromic, microcytic RBCs; normal leukocytes and platelets

CBC = complete blood count, g/dL = grams per deciliter, MCV = mean corpuscular volume, fL = Femtoliter, WBC = white blood cell, ×10^9^/L = times 10 to the power of 9 per liter, CRP = C-reactive protein, mg/L = milligrams per liter, ESR = erythrocyte sedimentation rate, mm/h = millimeters per hour, PT = prothrombin time, seconds = seconds, PTT = partial thromboplastin time, INR = international normalized ratio, D-dimer = D-dimer, ng/mL = nanograms per milliliter, RBCs = red blood cells, N/A = not applicable.

**Table 2:** Summary of metabolic and renal parameters, including iron status, kidney function, and supportive studies.

Parameter	Initial (day 1, local hospital)	Follow-up (day 4, our hospital)	Pediatric reference range	Comments
Iron studies				
Ferritin (ng/mL)	7.3	N/A	7–140	Low, consistent with iron deficiency anemia
Electrolytes				
Sodium (mmol/L)	141	139	135–145	Normal range
Potassium (mmol/L)	3.9	4.4	3.5–5.0	Normal range
Chloride (mmol/L)	111	103	98–107	Initially elevated, normalized at follow-up
Renal function				
Creatinine (mg/dL)	0.44	0.5	0.2–0.7	Normal range
Blood urea nitrogen (mg/dL)	10	26	5–18	Elevated in follow-up, possibly dehydration-related
Glucose				
RBS (mg/dL)	124	103	70–140	Normal range
Liver function tests				
AST (U/L)	45	N/A	10–50	Normal range
ALP (U/L)	314	N/A	100–350	Normal range
Muscle enzymes				
CK (U/L)	304	N/A	60–305	Upper normal range
Urinalysis				
WBC (/hpf)	N/A	1–2	<5	Normal range
RBC (/hpf)	N/A	1–3	<5	Normal range
Protein	N/A	Negative	Negative	Normal

/hpf = per high-power field, ALP = alkaline phosphatase, AST = aspartate aminotransferase, CK = creatine kinase, mmol/L = millimoles per liter, mg/dL = milligrams per decilitre, N/A = not applicable, ng/mL = nanograms per milliliter, RBC = red blood cell, RBS = random blood sugar, U/L = units per liter, WBC = white blood cell.

**Table 3:** Infectious, immunologic, and prothrombotic evaluations conducted to exclude systemic etiologies.

Parameter	Initial (day 1, local hospital)	Follow-up (day 4, our hospital)	Pediatric reference range	Comments
Infectious markers				
HBsAg	N/A	Negative	Negative	No evidence of hepatitis B infection
HCV	N/A	Negative	Negative	No evidence of hepatitis C infection
ASOT (IU/mL)	<200	N/A	<200	Normal
Rheumatologic studies				
ANA	N/A	0.52	<0.8 (negative)	Negative
Anti-dsDNA (U/mL)	N/A	7	<12 (negative)	Negative
c-ANCA (U/mL)	N/A	0.9	<5 (negative)	Negative
p-ANCA (U/mL)	N/A	1.1	<5 (negative)	Negative
Immunoglobulins (IgG/IgM/IgA)	N/A	Within normal range	Age-dependent	No abnormalities
Thrombophila profile				
Antithrombin III	N/A	123%	66%–124%	Within normal range for age
Protein S	N/A	97%	60%–120%	Within normal range for age
Protein C	N/A	86%	75%–135%	Within normal range for age
MTHFR	N/A	Negative	–	–
Homocystinemia	N/A	Negative	–	–
Leiden v	N/A	Negative	–	–
Anti-cardiolipin IGG	N/A	Negative	–	No evidence of anti-cardiolipin antibodies
Anti-cardiolipin IGG	N/A	Not Available	–	Test result unavailable
Anti-B2 glycoprotein IgM	N/A	Negative	–	No evidence of anti-B2 glycoprotein antibodies
Anti-B2 glycoprotein IgA	N/A	Negative	–	No evidence of anti-B2 glycoprotein antibodies
Anti-phospholipid IgG	N/A	Negative	–	No evidence of anti-phospholipid antibodies
Anti-phospholipid IgM	N/A	Negative	–	No evidence of anti-phospholipid antibodies

ANA = antinuclear antibody, anti-B2 glycoprotein IgA = anti-beta 2 glycoprotein immunoglobulin A, anti-cardiolipin IgG = anti-cardiolipin immunoglobulin G, anti-phospholipid IgG = anti-phospholipid immunoglobulin G, anti-phospholipid IgM = anti-phospholipid immunoglobulin M, ASOT = antistreptolysin O titer, c-ANCA = cytoplasmic antineutrophil cytoplasmic antibody, dsDNA = double-stranded DNA, HBsAg = hepatitis B surface antigen, HCV = hepatitis C virus, IgA = Immunoglobulin A, IgG = immunoglobulin G, IgM = immunoglobulin M, IU/mL = international units per milliliter, MTHFR = methylenetetrahydrofolate reductase, N/A = not applicable, p-ANCA = perinuclear antineutrophil cytoplasmic antibody, U/mL = units per millilitre.

Three days later, repeat laboratory testing at our center showed improved hemoglobin (11.06 g/dL) and MCV (69.12 fL). Inflammatory markers remained within normal range (ESR 10 mm/h; CRP 6 mg/L). Rheumatologic and thrombophilia panels were negative, and peripheral smear confirmed microcytic hypochromic anemia without abnormal cell morphology (Tables 1 and 3).

Initial Doppler ultrasound at the referring hospital revealed triphasic flow in the anterior and posterior tibial arteries but absent arterial signal in the plantar arch. Follow-up duplex imaging at our center demonstrated restored triphasic flow in the dorsalis pedis (DPA) and posterior tibial arteries with an arterial peak systolic velocity of approximately 90 cm/s. The plantar arch was patent. No evidence of vasculitis or thromboembolic occlusion was identified (Table 4).

**Table 4:** Imaging studies.

Timeline	Location	Findings	Comments
At initial referral	Referred Hospital	– Triphasic arterial flow in anterior and posterior tibial arteries	Normal proximal arterial flow, localized plantar arch compromise
		– No arterial occlusion or vasculitis	–
		– Absent arterial flow in the plantar arch of the right foot	Consistent with ischemic changes in the toes
2 d later	Our Tertiary Care Hospital	– Patent arterial and venous systems from groin to foot arch	Improved perfusion in follow-up imaging
		– Triphasic flow in dorsalis pedis (DPA) and posterior tibial (PTA) arteries. APSV ~90 cm/s	No evidence of vasculitis or systemic vascular pathology
		– Patent plantar arch	–

APSV = arterial peak systolic velocity, DPA = dorsalis pedis artery, PTA = posterior tibial artery.

A multidisciplinary team, including pediatric hematology, infectious disease, rheumatology, and vascular surgery, was involved in the management plan. The patient received subcutaneous enoxaparin (14 mg every 12 hours), low-dose aspirin (40 mg once daily), and intravenous antibiotics (cloxacillin and ceftriaxone) for 5 days. This was followed by a 5-day course of oral cefdinir (250 mg/5 mL; 4 mL daily). Clinical improvement was observed during hospitalization with gradual resolution of erythema and return of perfusion to the affected digits.

**Figure 2.**
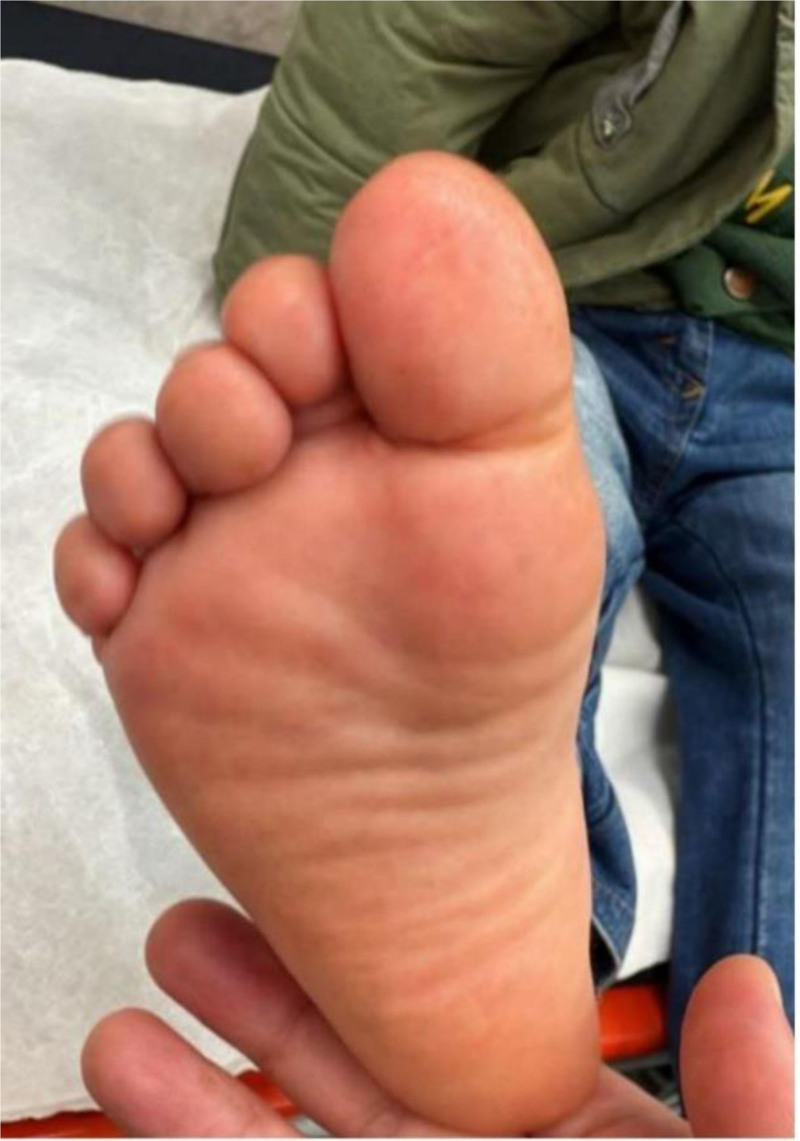
Photograph taken during follow-up demonstrates the plantar surface of the affected foot. The image shows normal skin coloration, with no evidence of ischemic changes, erythema, or residual discoloration, confirming complete recovery.

At discharge, the patient continued enoxaparin and aspirin with weekly outpatient follow-up. Complete resolution of ischemic discoloration was achieved within 30 days. No surgical intervention was required. At the 1-month follow-up visit, the foot appeared normal with no residual skin changes or functional deficits (Fig. 2).

## 3. Discussion

Acute digital ischemia in pediatric patients is an uncommon but potentially serious condition characterized by the sudden interruption of blood flow to 1 or more digits, leading to tissue discoloration, ischemia, and in severe cases, necrosis.^[10]^ The diagnosis in our case was supported by clinical findings of localized black discoloration and imaging evidence of absent flow in the plantar arch, with no signs of systemic inflammation or hypercoagulability.

While trauma, vasculitis, and systemic thrombotic disorders are common causes of digital ischemia in children,^[6]^ arthropod-related vascular injury remains a rare and underrecognized etiology. In our case, the suspected involvement of an arthropod was based on the mother’s observation of an insect near the affected foot. However, the specific arthropod was not captured or identified, which presents a significant limitation in confirming the etiology. The description provided (“cockroach ground beetle”) is nonscientific and likely inaccurate, highlighting the risk of relying on layperson identification. Therefore, the case emphasizes the importance of retrospective identification, using clinical features, exclusion of other causes, and progression of the condition to guide diagnostic reasoning.

Arthropod bites have been associated with vascular injury through various mechanisms, including venom-mediated endothelial damage, vasospastic reactions, and local inflammatory responses.^[7,8]^ For instance, *Loxosceles* spider venom has been shown to disrupt endothelial integrity, activate neutrophils, and trigger thrombosis.^[8]^ Although systemic envenomation by scorpions or spiders may present with diffuse vascular effects,^[11]^ our case represents a localized vascular insult without systemic manifestations.

From a management perspective, our patient benefited from early conservative intervention, including anticoagulation with enoxaparin,^[12]^ antiplatelet therapy with low-dose aspirin,^[13]^ and prophylactic antibiotics.^[14]^ This approach was guided by multidisciplinary consultation and aligns with previous pediatric studies demonstrating the safety and efficacy of nonsurgical management in ALI.^[3,9]^ Importantly, the patient improved without the need for invasive procedures or limb-threatening complications, illustrating the favorable prognosis with timely, coordinated care.

This case has several strengths. It adds to the limited literature on arthropod-induced ischemia in children and reinforces the importance of considering rare etiologies when common causes are excluded. It also underscores the utility of a multidisciplinary approach in pediatric vascular emergencies.

However, there are important limitations. The lack of direct identification or capture of the offending arthropod limits definitive causal attribution. Furthermore, the retrospective diagnostic process, while reasonable, relies heavily on clinical exclusion and remains inherently uncertain. Additional limitations include the absence of tissue biopsy or histopathology, which could have provided confirmatory evidence of envenomation-related vascular injury.

In conclusion, this case highlights the value of detailed history-taking, broad differential consideration, and prompt conservative treatment in managing acute digital ischemia in children. Arthropod-induced vascular injury, though rare, should be considered in otherwise unexplained presentations, particularly when localized findings and exclusion of systemic etiologies support such suspicion.

## 4. Conclusion

This case illustrates a rare instance of acute digital ischemia in a child, suspected to be associated with an arthropod-related vascular insult. It highlights the importance of including unusual etiologies, such as arthropod-induced vasospasm or endothelial injury, in the differential diagnosis of pediatric ischemic presentations. Early clinical suspicion, prompt conservative management, and multidisciplinary coordination were key to achieving full recovery without surgical intervention. Clinicians should remain vigilant for atypical vascular presentations and consider conservative approaches when appropriate.

## Acknowledgments

We would like to thank the patient and their family for consenting to the publication of this report. All individuals named in the acknowledgments have provided permission to be included.

## Author contributions

**Conceptualization:** Abdallah A. Najjar, Mahmoud Abdelrazzaq Abu Mayaleh, Mohamed Abdelrazzaq Abu Mayaleh.

**Data curation:** Abdallah A. Najjar, Mahmoud Abdelrazzaq Abu Mayaleh, Mohamed Abdelrazzaq Abu Mayaleh.

**Formal analysis:** Mahmoud Abdelrazzaq Abu Mayaleh.

**Investigation:** Mohamed Abdelrazzaq Abu Mayaleh.

**Project administration:** Roba Alzuhoor.

**Resources:** Abdallah A. Najjar, Mahmoud Abdelrazzaq Abu Mayaleh, Roba Alzuhoor, Fawzi Mazen Nejmah.

**Supervision:** Fawzi Mazen Nejmah.

**Validation:** Fawzi Mazen Nejmah.

**Visualization:** Fawzi Mazen Nejmah.

**Writing – original draft:** Abdallah A. Najjar, Mahmoud Abdelrazzaq Abu Mayaleh, Roba Alzuhoor.

**Writing – review & editing:** Abdallah A. Najjar, Mahmoud Abdelrazzaq Abu Mayaleh, Roba Alzuhoor, Mohamed Abdelrazzaq Abu Mayaleh, Fawzi Mazen Nejmah, Dalia Abu-Isneineh.
